# The Odd Faces of Oligomers: The Case of TRAF2-C, A Trimeric C-Terminal Domain of TNF Receptor-Associated Factor

**DOI:** 10.3390/ijms22115871

**Published:** 2021-05-30

**Authors:** Almerinda Di Venere, Eleonora Nicolai, Velia Minicozzi, Anna Maria Caccuri, Luisa Di Paola, Giampiero Mei

**Affiliations:** 1 Department of Experimental Medicine, Tor Vergata University of Rome, Via Montpellier 1, 00133 Rome, Italy; divenere@med.uniroma2.it (A.D.V.); nicolai@med.uniroma2.it (E.N.); 2 Department of Physics, Tor Vergata University of Rome, Via Della Ricerca Scientifica 1, 00133 Rome, Italy; velia.minicozzi@gmail.com; 3 Department of Chemistry, University of Rome Tor Vergata, Via Della Ricerca Scientifica 1, 00133 Rome, Italy; caccuri@uniroma2.it; 4 Unit of Chemical-Physics Fundamentals in Chemical Engineering, Department of Engineering, University Campus Bio-Medico of Rome, Via Álvaro del Portillo 21, 00128 Rome, Italy

**Keywords:** trimeric protein, protein-protein interface, protein clusters analysis

## Abstract

TNF Receptor Associated Factor 2 (TRAF2) is a trimeric protein that belongs to the TNF receptor associated factor family (TRAFs). The TRAF2 oligomeric state is crucial for receptor binding and for its interaction with other proteins involved in the TNFR signaling. The monomer-trimer equilibrium of a C- terminal domain truncated form of TRAF2 (TRAF2-C), plays also a relevant role in binding the membrane, causing inward vesiculation. In this study, we have investigated the conformational dynamics of TRAF2-C through circular dichroism, fluorescence, and dynamic light scattering, performing temperature-dependent measurements. The data indicate that the protein retains its oligomeric state and most of its secondary structure, while displaying a significative increase in the heterogeneity of the tyrosines signal, increasing the temperature from ≈15 to ≈35 °C. The peculiar crowding of tyrosine residues (12 out of 18) at the three subunit interfaces and the strong dependence on the trimer concentration indicate that such conformational changes mainly involve the contact areas between each pair of monomers, affecting the oligomeric state. Molecular dynamic simulations in this temperature range suggest that the interfaces heterogeneity is an intrinsic property of the trimer that arises from the continuous, asymmetric approaching and distancing of its subunits. Such dynamics affect the results of molecular docking on the external protein surface using receptor peptides, indicating that the TRAF2-receptor interaction in the solution might not involve three subunits at the same time, as suggested by the static analysis obtainable from the crystal structure. These findings shed new light on the role that the TRAF2 oligomeric state might have in regulating the protein binding activity in vivo.

## 1. Introduction

Protein complexes play fundamental roles in cells life. Oligomerization allows the fine tuning of allosteric enzymes [1,2], creates new active/binding sites [3], and speeds up consecutive reactions by substrate channeling [4,5]. The vast majority of protein oligomeric structures examined so far is represented by homomers, which are oligomers composed by identical subunits [6,7,8]. The reason for the prevalence of homomers (and, in particular, homodimers) arises from the enhanced interaction propensity displayed by monomers with similar surfaces [9]. This feature is due to statistical reasons and has been used by evolution to select a number of possible architectures that provides functional and structural advantages to complexes made by identical subunits [9,10]. Among them, homo-trimers are particularly intriguing: They are the simplest way in which a cyclic homomer can be assembled [11,12] and, at variance with homo-dimers, each subunit is involved in a head-to-tail configuration that forms two different interfaces with the other two monomers. They are widely distributed in a variety of living organisms acting, for instance, as viral infection mediators [13] or bacterial adhesines [14]. In humans, several kinds of membrane receptors are active as homo-trimers and some of them have been found to be strictly correlated to severe pathologies, such as cardiovascular diseases [15] or involved in cell death induction [16]. Noticeably, the spike proteins of SARS-CoV2, in charge for the high infectivity of the virus, is a nucleocapsid homo-trimeric protein [17].

**Figure 1:**
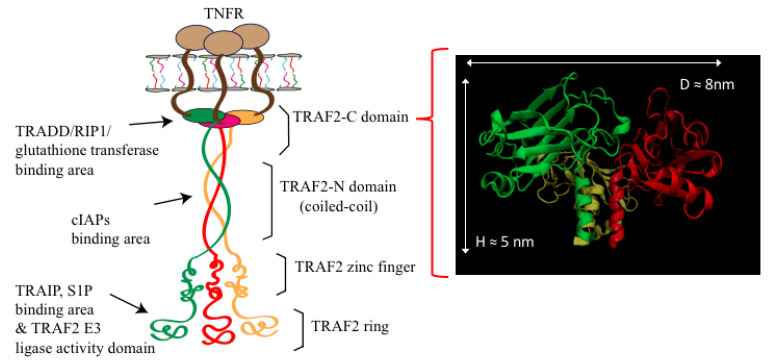
Left: Schematic representation of the TNFR-membrane-TRAF2 system. The receptor is sketched in brown, while the three TRAF2 subunits are shown in green, red, and orange. The protein domains and the main binding areas and ligand proteins are also indicated. Right: Crystallographic model of TRAF2-C is shown (pdb: 1CA4).

The receptors of the tumor necrosis factor (TNFRs) and their associated proteins (TRAFs) also belong to the class of homo-trimers. They have raised a great interest in the scientific community, being involved in the signaling pathways of cancer and inflammation [18,19]. In particular, the role of TRAF2 is to mediate the signal transduction, interacting with the receptors and with a number of other proteins including: (i) The TNFRs themselves [20]; (ii) The TRADD adaptor protein [21]; (iii) The homologous TRAF1 [22]; (iv) The glutathione transferase P1-1 [23]; (v) The cellular inhibitors of apoptosis, cIAPs, [22]; (vi) The zinc finger protein A20 [24]; (vii) The protein kinase RIP1 [25]. Thanks to such manifold binding capability, TRAF2 acts as a link between the receptor and the downstream effector proteins allowing a fine regulation of NF-kB MAPK signaling pathways [26]. The characterization of TRAF2 structure and dynamics is therefore crucial for understanding the signaling process in which it is involved. TRAF2 shares a common domain organization with other members of the TRAFs family, consisting of a globular carboxy-terminal domain (TRAF-C) and an extended coiled-coil section (TRAF-N) made up of three long alpha helices. Most of the principal sites of interaction with other proteins have been identified on the surface of the C-terminal domain and in the middle of the coiled-coil section (Figure 1).

The equilibrium between trimeric TRAF2 and its dimeric/monomeric species is crucial for the protein activities. The interaction between TRAF2 and TNRFs receptors occurs with both proteins in the trimeric form [20]. On the other hand, TRAF2 dimers form hetero-trimeric oligomers with TRAF1 (i.e., TRAF1: [TRAF2]_2_), which displays an enhanced propensity in cIAPs binding [22]. Finally, the protein C-terminal domain, TRAF2-C, strongly interacts with membranes when dissociated into monomeric/dimeric forms [27,28]. All these findings suggest that the oligomerization process might have a regulatory function on the TNF signaling in vivo. Recently, thanks to the analysis of pressure-induced dissociation measurements and to molecular dynamics (MD) simulation we have found that TRAF2-C probably exists in the solution as an “asymmetric” trimer, in which two subunits cluster together while the third becomes more independent from the others [29]. We have argued that this behavior must be strictly dependent on the interactions at the monomer-monomer interfaces, so in this paper we have applied dynamic light scattering, circular dichroism, and fluorescence spectroscopy to monitor the structure and conformational dynamics of TRAF2-C as a function of temperature. Furthermore, the protein dynamics have been simulated in silico at two different temperatures in the pre-denaturation range and the outputs analyzed by a protein contact network approach [30]. The results demonstrate that in order to reach the physiological temperature (≈35 °C) few, critical hotspot areas of the trimers interfaces govern the monomer-dimer-trimer equilibrium and the time-dependent asymmetric approaching and distancing of the subunits. These dynamics also influence the roughness of the protein external surface, opening new points of view on the way TRAF2 binds to TNRF receptors.

## 2. Results

### 2.1. Insights on TRAF2-C Aggregation State and Secondary Structure Content at Different Temperatures

**Figure 2:**
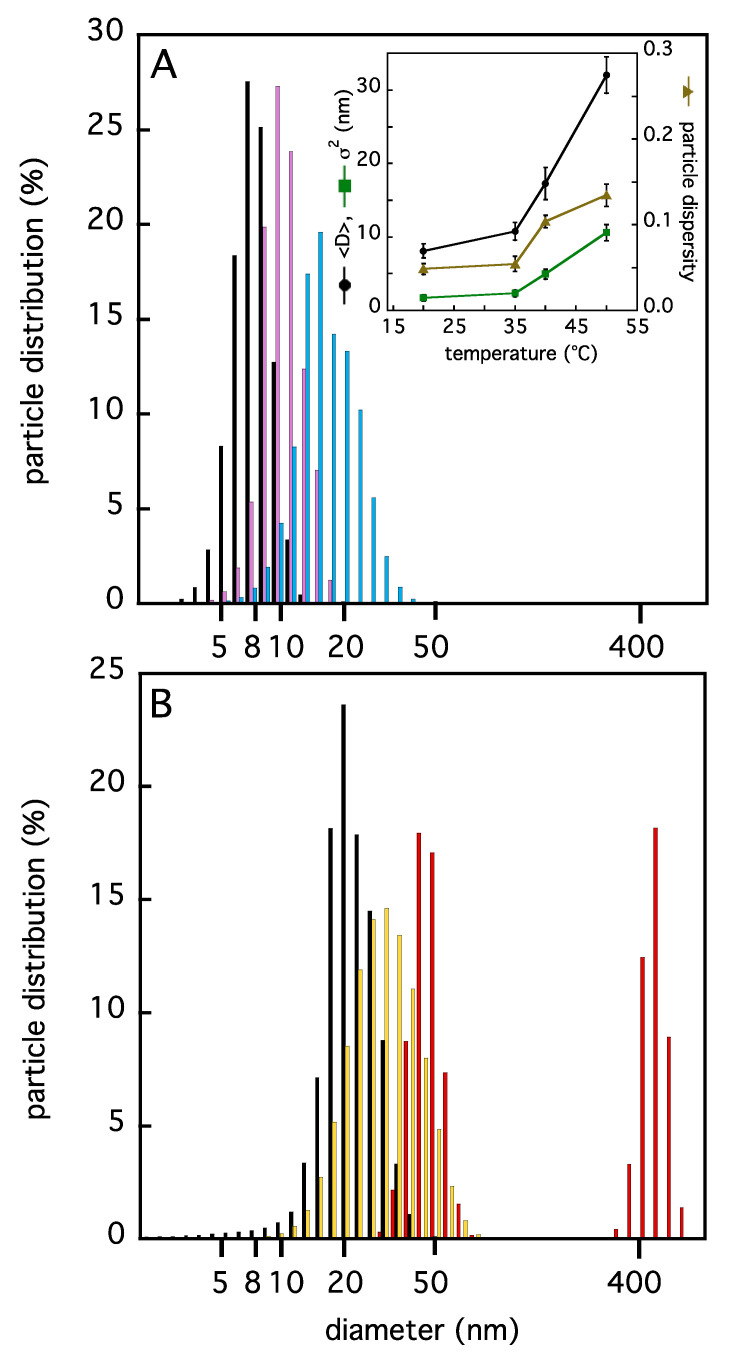
Particle size distributions of TRAF2-C (4 μM in KB) as a function of temperature from DLS measurements. In panel (**A**), the distributions at 20, 35, and 40 °C (black, purple, and cyan bars, respectively), are reported. In panel (**B**), the results at 50 °C (yellow bars) and 60 °C (red bars) are shown. The return at 20 °C (black bars) is also reported to demonstrate irreversibility. In the inset of panel A the values of the average diameter, <D> (black circles), and the variance, σ^2^ (green squares) of Gaussian distribution data fits are shown. The dispersity (see Materials and Methods) of the samples at different temperatures is also reported (brown triangles).

Dynamic light scattering measurements have been carried out at different temperatures to assess the protein oligomeric state. The average size of the experimental hydrodynamic diameter obtained, <D>, is reported in Figure 2, in the range 20–60 °C. At low temperatures (T ≤ 35 °C) the particle distribution is sharp and mono-disperse, as indicated by an estimation of the particle dispersity (Figure 2A, inset). The average TRAF2-C diameter at 20 °C results to be ≈ (8.1 ± 0.9) nm. The theoretical diameter of a perfect sphere with an equivalent molecular weight (≈3 × 20,000 = 60,000) is ≈6 nm [31,32], while the hydrated radius of the trimer, estimated in silico through MD simulation at 37 °C, is R_H_ ≈ 4.7 nm (see Materials and Methods).

The larger size obtained in the light scattering measurements (Figure 2A) is compatible with the real protein tridimensional assembly. In fact, according to the crystallographic model, TRAF2-C has a mushroom-shaped structure whose globular head has a diameter in the range 5–8 nm, while the length of the coiled-coil tail has an extension of about 5–6 nm ([33] and Figure 1). As a matter of fact, previous measurements of TRAF2-C diffusion coefficient at 25 °C through fluorescence correlation spectroscopy [32] yielded for the trimer ≈6.9·10^−11^ m^2^/s, which corresponds to a hydrodynamic radius of about ≈3.6 nm. A good data reversibility from 35 °C was achieved restoring the initial condition, since a progressive narrowing and shift to smaller diameter values was obtained, with a particle distribution superimposable to that at 20 °C (data not shown).

From T ≈ 40 °C a significant widening of the particle distribution occurs, accompanied by a shift towards larger <D> values (Figure 2A,B) diagnostic of an increased sample heterogeneity (Figure 2A, inset). Above 40 °C, reversibility is lost (Figure 2B) and a relevant molecular aggregation process takes place, as demonstrated by the presence of a second, long component at 60 °C.

**Figure 3:**
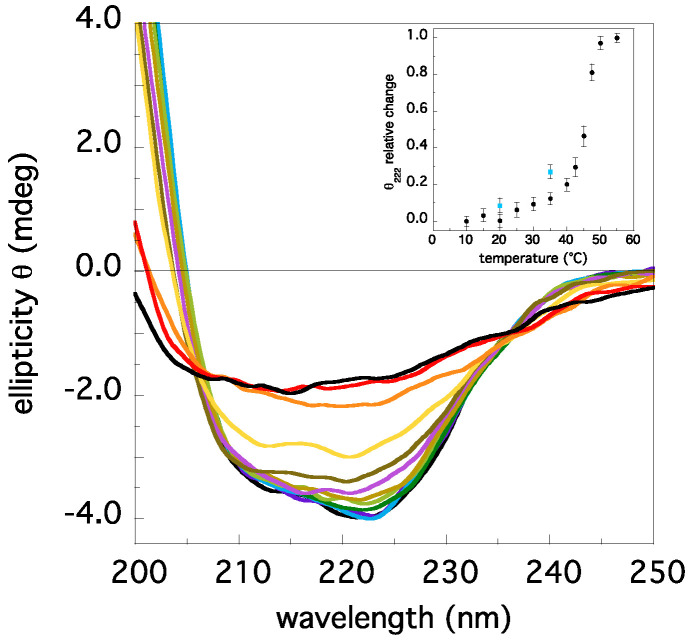
Circular dichroism spectra at different temperatures (10 °C, black; 15 °C, purple; 20 °C, cyan; 25 °C, dark green; 30 °C, light green; 35 °C, light brown; 40 °C, light purple; 42.5 °C, dark brown; 45 °C, yellow; 47.5 °C, orange; 50 °C, red; 55 °C, black), at the protein (monomers) concentration of 2 μM. In the inset, the relative change in the ellipticity at 222 nm is reported. The filled circles in cyan correspond to the ellipticity measured for a sample ten times less concentrated (0.2 μM).

Circular dichroism spectroscopy measurements have been used to monitor the protein secondary structure content at increasing temperatures. The spectra in the peptidic region exhibit minor changes in the range 10–35 °C (Figure 3). An analysis of the signal at 222 nm (Figure 3, inset) reveals a loss of ≈−10% in the signal, around 35–40 °C. This effect is protein-concentration dependent (Figure 3, inset), suggesting the occurrence of partial dissociation into species with lower molecular weight (monomers/dimers), as the temperature increases. In this range, almost all the initial signal intensity (≈+98%) can be restored returning at T = 10 °C, while reversibility is lost above T = 40 °C (data not shown). In a previous characterization of TRAF2-C stability and folding we demonstrated that guanidine-induced monomerization produced monomers with an overall native-like tertiary structure and with a reduced secondary structure content (≤−40%, [32]). Since in a MD simulation at 25 °C monomeric subunits exhibit an increased mobility of the N-terminal alpha-helix section [32], we might conclude that the loss of the CD signal at 222 nm (Figure 3, inset) might arise from two different effects: (i) A partial de-structuration of the trimers; (ii) An increase of the monomers (and dimers) content. This finding allows performing also a raw esteem of the protein oligomerization state at 37 °C. According to the dissociation constant obtained at 20 °C, K_d_ ≈ 1.4·10^−16^ M^2^ [32], the percentage of trimers when the protein (monomers) concentration is 2 μM is around ≈90%. If the CD change (at 37 °C) observed in Figure 3 (−10%) were ascribed to the sole monomerization, the maximum increment in the monomers content at 37 °C could be expected, of the order of +20%.

### 2.2. Analysis of TRAF2-C Tertiary Structure and Temperature-Induced Conformational Changes by Fluorecence Spectroscopy

**Figure 4:**
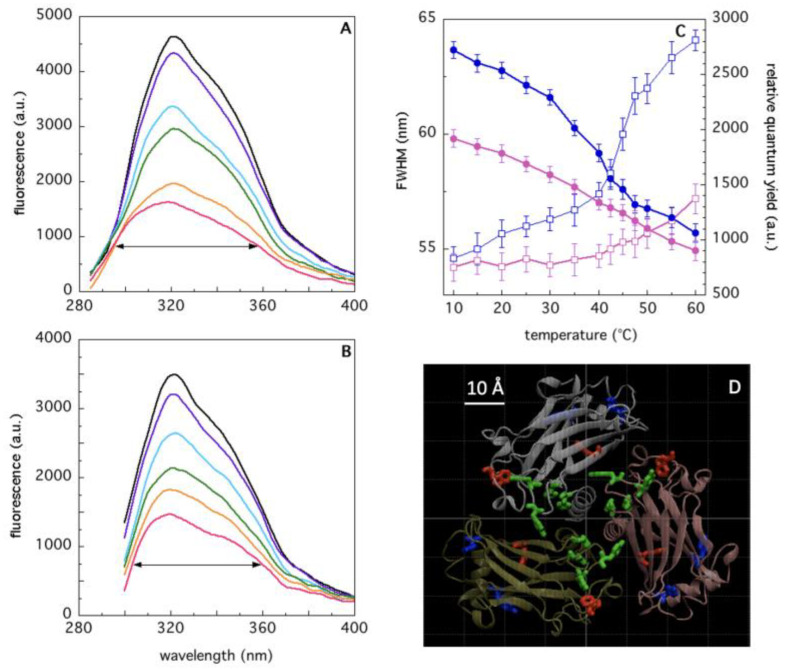
Fluorescence spectra of TRAF2-C as a function of temperature (10 °C, black; 20 °C, purple; 35 °C, cyan; 40 °C, green; 50 °C, orange; 60 °C, red) upon excitation at 275 nm (panel (**A**)) and 292 nm (panel (**B**)). The relative fluorescence quantum yield (i.e., the total spectral area) and the full width at half maximum (FWHM), black horizontal arrows, panels (**A**,**B**)) are reported as a function of temperature in panel (**C**) (λ_ex_ = 275, blue lines; λ_ex_ = 292, red lines; filled symbols, quantum yield; empty symbol, FWHM). The position of the TRP residues (W356, W424 in red), of the TYRs close to the monomers interfaces (Y350, Y382, Y386, Y388, in green) and of TYRs located in the protein peripheral area (Y395, Y484, in blue) are reported in panel (**D**), in which a top view of trimeric TRAF2-C is shown.

TRAF2-C intrinsic fluorescence mainly arises from eight aromatic residues contained in each monomeric subunit, namely two tryptophan residues (W356 and W424) and six tyrosines (Y350, Y382, Y386, Y388, Y395, Y484). As a consequence, the shape of the protein fluorescence emission depends on the excitation wavelength used. Figure 4A reports that the spectrum of the whole set of intrinsic fluorophores is reported as a function of temperature (excitation wavelength λ_ex_ = 275 nm). In this condition, at room temperature (T = 25 °C), a strong energy transfer process is known to take place [32], from tyrosine to tryptophan residues. Increasing the temperature up to 35–40 °C results in a progressive, linear broadening of the spectrum profile upon excitation at 275 nm (Figure 4C, FWHM, blue empty squares), due to the appearance of a shoulder around 310 nm (Figure 4A). This effect (diagnostic of tyrosine emission) indicates an increased spectral heterogeneity (i.e., a larger number of emitting species), which reflects the temperature-induced perturbation of the microenvironment surrounding the tyrosine residues. In fact, the spectra of the sole tryptophans (obtained with λ_ex_ = 292 nm) do not present any relevant effect in the FWHM, up to 40 °C (Figure 4B). At higher temperature values (T ≥ 40 °C) a steeper transition occurs at both excitation wavelengths (Figure 4C), which parallels the loss of secondary structure (Figure 3) and molecular aggregation (Figure 2).

**Figure 5:**
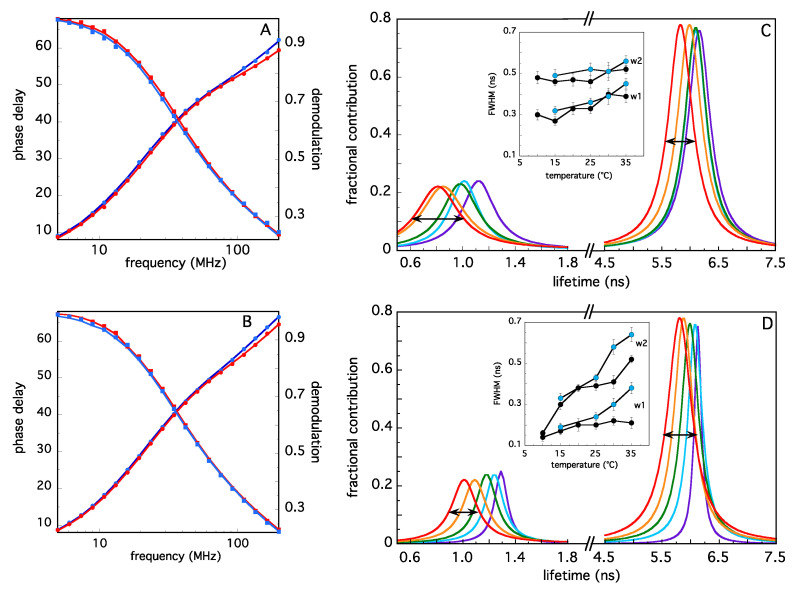
Phase shift (circles) and demodulation data (squares) of TRAF2-C (2 μM) at 20 °C (blue) and 37 °C (red) as a function of light excitation frequency ((**A**), λ_exc_ = 300 nm; (**B**), λ_exc_ = 280 nm). The solid lines represent the best fit obtained using the continuous distribution of lifetimes reported in panels C (λ_exc_ = 300 nm) and D (λ_exc_ = 280 nm) as a function of temperature (10 °C, purple; 15 °C, cyan; 20 °C, green; 30 °C, orange; 35 °C, red). The full FWHM (black horizontal arrows) at each temperature are reported in the insets of panels (**C**,**D**), at two different concentrations, namely 2 μM (black symbols) and 0.2 μM (cyan symbols).

The change in the TRAF2-C fluorescence lifetimes provides a more detailed characterization of temperature-dependent effects in the pre-denaturation range (i.e., 10 °C ≤ T ≤ 35 °C). The dynamic fluorescence of TRAF2-C is complex: Due to the presence of several aromatic residues in each subunit two continuous distributions of lifetimes (rather than a few discrete exponentials) are required to fit the data [28,32]. Figure 5 reports two examples of raw data (phase delay and demodulation as a function of excitation frequency, panels A and B) at 20 °C (blue symbols) and 37 °C (red symbols). The best fits (obtained through the two components of lifetimes reported in panels C and D) are shown as solid lines. The results of the fits obtained at five different temperatures and at two different excitation wavelengths (λ_ex_ = 300 nm, Figure 5C and λ_ex_ = 280 nm, Figure 5D) are reported in different colors. In the first case, at both low and high protein concentration, the quenching due to the temperature increase (i.e., the shift to shorter lifetime values) is accompanied by a modest change in the widths of both distributions (Figure 5C and inset).

On the contrary, the results upon excitation at 280 nm demonstrate a large increase in the heterogeneity of both components, when the TRAF2-C concentration (expressed in monomers) is [TRAF2-C]_M_ ≈ 0.2 μM (Figure 5D and inset), while a less pronounced effect is obtained at [TRAF2-C]_M_ ≈ 2 μM. These findings are in line with the results obtained by CD spectroscopy (Figure 3, inset) and indicate that, around 35 °C, the equilibrium among monomers, dimers, and trimers is severely controlled by both protein concentration and temperature changes.

### 2.3. In Silico Modeling of TRAF2-C Structural Dynamics and Cluster Analysis

**Figure 6:**
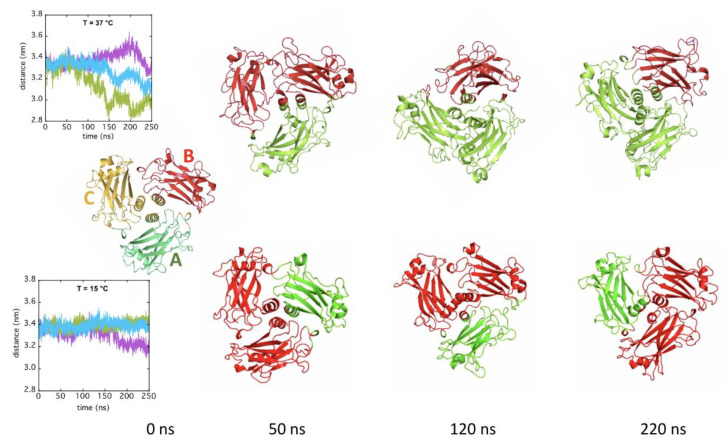
Network clustering on MD simulation frames as a function of time (0, 50, 120, 220 ns) at 37 °C (upper row) and 15 °C (lower row). The initial configuration in the crystal is reported for comparison (on the left, t = 0 ns) and the three subunits are indicated as A (green), B (red), and C (yellow). All the other cartoons have been re-oriented according to the X-ray configuration (t = 0 ns) for better clarity. Monomers belonging to the same cluster appear in the same color. In the insets, the distance between the center of mass of each couple of monomers (AB, purple; BC, cyan; CA, green) is shown.

The reciprocal movements of TRAF2-C subunits has been reproduced in silico by MD simulation. In a recent study [29], we have already analyzed the dynamics of TRAF2-C at 37 °C, which demonstrated how two subunits get progressively close as a function of time (Figure 6, upper inset). A protein contact network approach has demonstrated that at 220 ns they form a cluster (AC, Figure 6, last cartoon to the right in the upper row), while the third subunit (B) acts as a more “independent” ensemble. Here, we have extended such analysis to all the frames collected during the reproduction of the trimer dynamics and we have observed that such behavior begins since the very early steps of the simulation and that it is temperature dependent. The cartoons reported in Figure 6 correspond to the 2:1 clusterization analysis as a function of time, at two temperatures, namely 37 and 15 °C.

**Figure 7:**
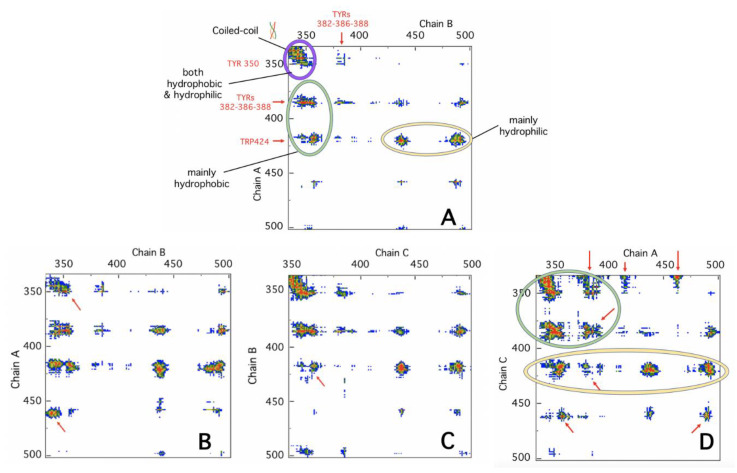
In panel A, the bi-dimensional interaction map of chain A and B in the crystallographic file is shown (couples (**B**,**C**) and (**A**,**C**) produce identical patterns). The red, yellow, green, and blue spots correspond to different distance ranges (7, 10, 13, 16 Å, respectively). The diagrams in the lower panels (**B**–**D**) report the main contacts of the three interfaces obtained by MD simulation at 220 ns, 37 °C. The red arrows identify the major changes with respect to time t = 0. The green, yellow, and purple ellipses group as well as the hydrophobic, hydrophilic, and mixed kinds of interactions are grouped, respectively. The position of the tyrosines and of tryptophan 424 close to the dimeric interface are indicated in red. The analysis was performed using the COCOMAPS software [34].

The results disclose the dynamic aspect of the TRAF2-C monomers association: At lower temperatures, the reduced distancing of the subunits (Figure 6, lower inset), promotes more frequent couple interchanges (AC, CB, AB), while at 37 °C the clustering of two monomers (A and C in the example reported) becomes prevalent, after 100 ns (Figure 6). In order to check the correspondence between the structural dynamics reproduced in silico and the outcomes of the spectroscopic measurements, we have analyzed the changes in the monomer-monomer interfaces contacts as a function of time. Figure 7A shows the most relevant contacts at the interface of a couple of monomers (AB) of the crystallographic file. The position of the buried TRP424 and of the four tyrosines lying at the two subunits interfaces is shown for each chain. The section of amino acids involved in the coiled-coil tail is also indicated. From the high density of red spots it is clear that four main sections in the protein sequence play the most relevant roles in stabilizing the dimeric interaction: (i) The whole coiled-coil N-terminal tail; (ii) The amino acids between position 380 and 390, which include three of the tyrosines located at the interface; (iii) TRP 424 and its nearest-neighbor residues; (iv) The amino acids close to position 490. The evolution of such network topology has been monitored as a function of time and, as an example, the bi-dimensional contact maps of the three couples of monomers (AB, BC, and CA) are reported in Figure 7 (panels B–D).

**Figure 8:**
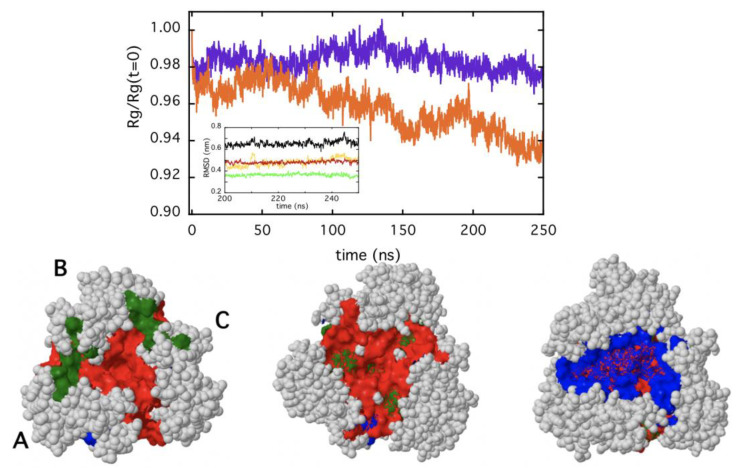
Upper panel: Relative gyration radius of TRAF2-C (with respect to the value at t = 0) obtained in the MD simulation at two temperatures, namely T = 15 °C (purple) and T = 37 °C (orange). In the inset, the root mean square deviation (RMSD) at 37 °C, for the last 50 ns of simulation is reported, for trimeric TRAF2-C (black line) and for the three subunits (A, green; B, yellow; C, red). Lower panel: The cartoons represent the main surface cavities distribution (top view) of trimeric TRAF2-C at three different times (t = 0, left; t = 50 ns, middle; t = 220 ns, right) of the molecular dynamics simulation performed at 37 °C. The most relevant surface cavities (in size and depth descending order) are reported in red, green, and blue, respectively. The analysis was performed using the 3D-surfer 2.0 software [35].

The formation of the dimeric cluster AC (around t ≈ 100 ns) and its further stabilization (at 220 ns) is particularly interesting. As shown in Figure 7D, the intensity of the crystallographic hot spots (in red) is considerably higher explaining the tight association nature of this specific cluster. Furthermore, new strong, hydrophobic interactions (indicated by the red arrows) appear and concur to the stabilization of this interface. In particular, the inter-chain interactions at the coiled-coil protein tail display an increased hydrophobicity in cluster AC (Figure 7D), becoming instead less crucial for the stabilization of the AB pair (Figure 7B). Such asymmetric behavior has a relevant impact on the structure of the trimer. In Figure 8, the change of the trimer gyration radius with respect to the initial conformation is reported as a function of the time, at two temperatures (15 and 37 °C). The RMSD of the last 50 ns at 37 °C is also reported (Figure 8, inset), to demonstrate that the equilibrium is reached only at the last part of the simulation. The reduction of the gyration radius (more evident at 37 °C) suggests a progressive increase in the compactness of the trimer due to the relative movements of the three subunits. Furthermore, an analysis of the protein structure (at 37 °C) demonstrates that another important consequence of such dynamics is a decrease in the roughness of the external surface (Figure 8, lower panel). In fact, as shown in the cartoons, the number and depth of the surface cavities decreases during the simulation time.

### 2.4. Simulation of TRAF2-C Binding to Peptide Receptor through Molecular Docking

**Figure 9:**
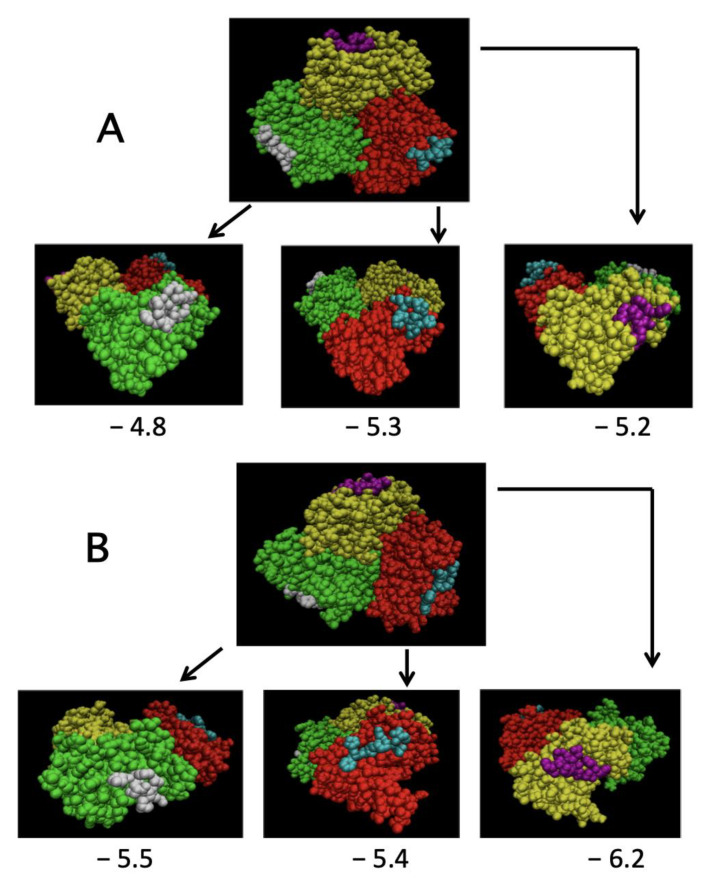
Results of the docking simulation of a TNF-R2 peptide to trimeric TRAF2-C at times t = 0 ns (panel (**A**)) and t = 220 ns (panel (**B**)). In each case, a top view (single frame) and the side view for each subunit are reported. The number shown under each picture represents the binding energy expressed in kcal/mol.

The crystallographic model of TRAF2-C has demonstrated that peptides from the TNFR family members can be used to simulate the protein binding to the trimeric receptors [20,36]. The crystallographic data have provided evidence that a conserved binding mode exists, allowing TRAF2 to recognize very different receptor sequences [20]. Taking advantage of these results, we performed some docking simulations using the TNF-R2 peptide (PFSKDDC) [33] in order to asses to which extent the surface structural effects due to monomers clustering (Figure 6, Figure 7 and Figure 8) might affect the binding of TRAF2-C to the receptor. In particular, the molecular dynamics frames at 37 °C have been used to find the best binding position for the peptide on each monomer, according to the maximum complex stability. Figure 9 reports results obtained at the beginning (t = 0 ns) and at the end (t = 220 ns) of the simulation. The positions occupied by the peptide on the initial crystallographic structure are symmetric and display binding energy in the order of 5 kcal/mol. On the contrary, the different overall protein surface obtained at 220 ns forces the peptide to bind to each monomeric subunit in a different way, with negative binding energy changes in the range 2–19%, thus suggesting that, in the solution, the clusterization process might indeed influence the association of TRAF2-C with TNFR receptors. In particular, despite the fact that both proteins interact in vivo as homotrimers, it is tempting to speculate that their binding might not necessarily occur in a symmetric way, unlike what the sole crystallographic structure information initially suggested [20].

## 3. Discussion

In the last decade, the study of protein interfaces in oligomers has led to important discoveries on the manifold functional roles and evolutionary advantages that the association of several monomeric subunits provides in several aspects of cell life (structure, metabolism, defense, etc.). One striking feature of protein interfaces is the large number of hydrophobic residues that typically populate these contact areas [10] and that mutually interact through a large number of weak forces, principally represented by van der Waals interactions [37]. Aromatic residues are particularly abundant at interfaces, participating in both inter-chain and intra-chain interactions [38]. Thanks to their hydrophobic propensity, their rings can stabilize the subunits complex [39], being at the same time important for protein-protein recognition [40]. The analysis of TRAF2-C crystal structure demonstrates that two phenylalanines (F354 and F491) from one subunit and two tyrosines (Y350 and Y386) from another are located at interfaces. Moreover, F381, Y382, Y388, Y395, Y484, and W424 are also very close to the contact area of each couple of monomers (Figure 4D). Such special configuration makes intrinsic fluorescence a suitable tool to monitor TRAF2-C conformational changes at the monomers interface. Steady-state and dynamic fluorescence measurements demonstrate that the protein structural heterogeneity increases, raising the temperature from 10 to 60 °C, and that two different kinds of events occur, being T ≈ 40 °C the transition threshold that discriminates between them. Above this temperature, the loss of CD signal and the DLS patterns indicate that the protein dramatically loses its secondary structure (Figure 3) and is liable to irreversible aggregation (Figure 2). On the contrary, more interesting structural information are obtained in the low temperatures range. In particular, between 10 and 35 °C, the more relevant spectroscopic effects are observed using an excitation wavelength of about λ_ex_ ≈ 275–280 nm (Figure 4 and Figure 5), thus suggesting that structural changes occur within the local tyrosines micro-environment and, most likely, at the monomers interface. This hypothesis is supported by the dependence of the lifetime distributions on the TRAF2-C concentration (Figure 4B, inset), that indicates the occurrence of subunits dissociation as the temperature approaches 35–40 °C. Previous measurements and simulations have demonstrated that monomeric TRAF2-C retains the globular structure of the C-terminal domain in the trimer, the only change being a more disordered α-helix segment, located at the protein coiled-coil tail [32]. Indeed, the analysis of the final simulation frames at 37 °C demonstrates that this domain contains the largest number of interface interactions and plays a major role in the formation of dimeric clusters (Figure 7B–D). Therefore, such propensity of TRAF2-C to form an asymmetric 2:1 complex could be an intrinsic, dynamic structural property of the trimer that, at a physiological temperature, might eventually dissociate into dimeric and monomeric species. This is evidently in contrast with the symmetric arrangement reported in literature [33], despite the fact that some clues of a possible protein asymmetric behavior in the solution emerge also from the X-ray crystallographic results. In fact, the unit cell contains two trimers and only two protomers out of six bind model receptor peptides [33]. The asymmetric distancing of the subunits observed in the pre-denaturation range (15–35 °C) has relevant structural consequences since it can facilitate dissociation, leading above 40 °C to aggregation and irreversible unfolding.

The biological relevance of such structural feature of TRAF2-C is straightforward: One main functional role of TRAF2 is to bind specific cellular inhibitors of apoptosis (cIAP proteins), in order to mediate in vivo TNF signaling [41]. However, the binding to cIAPs preferentially takes place in a hetero-trimeric form of TRAF2: Crystallographic and in solution measurements have, in fact, demonstrated that TRAF2 form oligomers with a homologous member of the same protein family (namely TRAF1), yielding a [TRAF1: (TRAF2)_2_] complex [22]. The docking simulations reported in Figure 9 suggest also a further, important consequence of the dynamic conformational fluctuations that take place at the monomer-monomer interface: The conformational drift that initiates at the center of the trimer and leads to dimeric clusterization (Figure 6), propagates to the protein surface and affects the binding to TNRF receptors peptides, thanks to an asymmetric configuration. At this regard, the dependence of TRAF2 oligomerization on the length of its coiled-coil portion deserves to be described in better details, finding the condition (ionic strength, pH, etc.) in which longer constructs are stable.

Whether dynamic asymmetry is a specific feature of TRAF or might be rather generalized to other homo-trimeric proteins, due to their unique subunits arrangement, is an interesting question. Certainly, the recent finding of the 2019-nCoV spike protein also adopts a dynamic asymmetric conformation to better interact with its receptor [42], is definitely a good starting point, considering that this feature might be crucial for its allosteric regulation [43] and might help improve the efficacy of serological tests [44,45].

## 4. Materials and Methods

### 4.1. Sample Preparation

TRAF2-C was expressed in *E. coli* BL21 and purified as previously reported [27]. Briefly, *E. coli* BL21 (DE3) cells were transformed with the His-tagged human TRAF2-C domain (residues 310–501) construct. After TRAF2-C expression, cells were lysed in a lysis buffer (LB, 20 mM Tris-HCl pH 8.0, containing 150 mM NaCl, 20 mM imidazole, 10% glycerol, 1 mM DTT, and EDTA-free inhibitor of protease). The cellular extract was loaded on a 10 mL NiNTA column pre-equilibrated with LB and the protein was eluted using a linear imidazole gradient. Imidazole was then removed from the TRAF2-C sample by filtration through a Sephadex G25 column (GE Healthcare Life Science, Chalfont St. Giles, UK) pre-equilibrated with the kinase buffer (20 mM Tris-HCl pH 7.6 containing 150 mM NaCl and 10% glycerol). TRAF2-C was stored in a kinase buffer and used in the same buffer during all spectroscopic measurements.

### 4.2. Spectroscopy Measurements

The protein oligomeric state was monitored using the light scattering technology. Measurements were performed on a Horiba (Kyoto, Japan) LB-500 nanoparticle size analyzer, equipped with a 650 nm, 5 mW laser diode. Data analysis was carried out using the accompanying software based on a Fourier-transform deconvolution procedure. We evaluated the heterogeneity of the distribution obtained through DLS measurements in two different ways. First, we obtained the variance of the distribution as a function of temperature fitting the data sets collected at 20, 35, 40, and 50 °C with a Gaussian-shaped function, which also yielded the value of the average particle diameter (Figure 2A, inset). Then, we evaluated the particle dispersity using (by analogy with the mass dispersity function using polymer science) the expression:

(1) $$\mathrm{particle}\;\mathrm{dispersity}=\frac{\sum_{i}{\left(D-\langle D\rangle \right)}^{2}q\left(i\right)/100}{{\langle D\rangle }^{2}}$$

where $\langle D\rangle =\sum_{i}D_{i}\;q\left(i\right)/100$, the q(i) being the experimental DLS distribution.

Steady-state fluorescence spectra were collected on a K2 spectrofluorometer (ISS, Champaign, IL, USA), thermostating the sample with an external bath circulator and checking the temperature through a thermocouple. Dynamic fluorescence data were obtained using the phase-shift and demodulation technique on a K2 spectrofluorometer (ISS, Champaign, IL, USA), equipped with laser diodes as excitation sources (λ_1ex_ = 280 nm and λ_2ex_ = 300 nm). Emission was observed through the WG 305 and WG 320 cutoff filter to avoid light scattering. The phase delay and the demodulation of the intrinsic TRAF2-C fluorescence signal with respect to a standard reference (p-terphenyl in ethanol) as a function of excitation frequency were fitted using a discrete exponential decay function (up to 4 discrete components) or a continuous, Lorentzian shaped distribution of lifetimes, using the ISS software. The continuous distribution model resulted in being more suitable to fit the data, on the basis of the minimum chi-squared value and the residues distribution profile, as already observed in previous measurements [28,32].

CD spectra were collected on a Jasco-710 spectropolarimeter in the range 200–250 nm using the quartz cuvette with a path length of L = 0.1 cm and thermostating the sample holder by an external water bath.

### 4.3. Molecular Dynamics

The classical MD at 288 K was performed with exactly the same parameters and the same procedure followed in [29] for the simulation at 310 K. An estimation of the TRAF2-C hydrodynamic radius has been obtained from MD, evaluating the protein diffusion coefficient, D, from the standard deviation:

(2) $${\left(r_{i}\left(t\right)-r_{i}\left(0\right)\right)}^{2}=6Dt$$

which yielded at 37 °C:

(3) $$D≅7\cdot 10^{-11}\;m^{2}\cdot s^{-1}.$$

The hydrodynamic radius, R_H_, can be obtained from the Einstein equation:

(4) $$D=\frac{k_{B}T}{6\pi \eta R_{H}}$$

where $\eta =6.9\cdot 10^{-4}\;Pa\cdot s$ is water viscosity at 37 °C, yielding R_H_ ≈ 4.7 nm.

Blind docking calculations were carried out using the Autodock4.2.6 (ADT) suite of codes [46,47]. The code combines two conformational research methods: The genetic algorithm and the local research. For each protein conformation, we generated 100 poses for the ligand (the TNF-R2 peptide allowed to be flexible) using 25 × 10^6^ steps of genetic algorithm and 300 steps of local search. The ADT calculates maps (or grids) of interaction considering the different ligand and receptor atom types through the definition of a cubic box. Then, for each grid ADT calculates the interaction energies that express the affinity of the ligand for the receptor. The space explored in the in silico docking was between 2·10^5^ and 2·10^6^ Å^3^, using a grid of 126 × 126 × 126 points, equispaced between 0.375 and 1Å.

### 4.4. Protein Contact Networks

The protein contact networks are built upon molecular dynamics frames (pdb files), according to the methods widely explained in a previous paper [48]. In brief, the alpha-carbons positions are extracted and the matrix of mutual distances between residues is computed. The network nodes are the single residues and a link exists between two of them if their distance falls between 4 and 8 Å, to include only noncovalent intramolecular interactions (considering that the peptidic bond length is around 3.5 Å). Indeed, a recent work [49] demonstrates that the optimal (single) cutoff is around 5 Å, which falls well in the range we proposed. The adjacency matrix *A* is the mathematical descriptor of the protein contact network, it is a binary matrix whose generic element is one if there is a link between the corresponding nodes. According to the adjacency matrix, the node degree reports the number of links it participates in. The degree matrix *D* is a diagonal matrix whose non null (diagonal) elements are the node degrees.

The network clustering is based on the spectral decomposition of the Laplacian matrix *L* [50] defined as:

(5) L=D−A

We used the eigenvector corresponding to the second minor of eigenvalue *v2* (Fiedler vector), which is of interest for the network partition in two clusters: The nodes fall in one cluster or another according to the corresponding sign of the *v2* components.

## Data Availability

Data is contained within the article.

## Abbreviations

MDPI	Multidisciplinary Digital Publishing Institute
DOAJ	Directory of open access journals
MD	Molecular Dynamics
CD	Circular dichroism
DLS	Dynamic light scattering
TNF	Tumor necrosis factor
TNFR	Tumor necrosis factor receptor
TRAF(s)	TNF receptor-associated factors
RMSD	Root mean square deviation
TRAF2-C	C-terminal domain truncated form of TRAF2
